# Prevalence and associated factors of human haemorrhagic fevers in Senegal: a comprehensive analysis of Hantaan, Crimean-Congo haemorrhagic fever and Rift Valley fever

**DOI:** 10.3389/fpubh.2025.1745257

**Published:** 2026-01-12

**Authors:** Maryam Diarra, Safietou Sankhe, Mamadou Aliou Barry, Fatoumata Diene Sarr, Mamadou Korka Kindy Diallo, Joseph Faye, Mame Astou Gassama, Maimouna Mbanne, Ousmane Faye, Cheikh Talla, Robab Katani, Keersten Ricks, Moussa Moise Diagne, Jessica Radzio-Basu, Cheikh Loucoubar

**Affiliations:** 1 Department of Epidemiology, Clinical Research and Data Science, Institut Pasteur de Dakar, Dakar, Senegal; 2 Department of Virology, Institut Pasteur de Dakar, Dakar, Senegal; 3 Public Health Direction, Institut Pasteur de Dakar, Dakar, Senegal; 4 Digital Direction and Information Systems, Institut Pasteur de Dakar, Dakar, Senegal; 5 The Huck Institute of the Life Sciences, Pennsylvania State University, State College, PA, United States; 6 Diagnostic Systems Division, United States Army Medical Research Institute of Infectious Diseases, Frederick, MD, United States

**Keywords:** associated factors, Crimean-Congo haemorrhagic fever, cross-sectional survey, hantavirus, Rift Valley fever, Senegal, seroprevalence

## Abstract

**Introduction:**

Viral haemorrhagic fevers such as Rift Valley fever, Crimean-Congo haemorrhagic fever, and hantavirus disease continue to threaten public health in Africa. This study assessed the seroprevalence and associated factors of these infections in Senegal.

**Methods:**

A cross-sectional survey was conducted from September 2022 to June 2024 among asymptomatic individuals living in close contact with livestock in two regions: Matam, a transboundary area; and Thiès, a non-transboundary area with high livestock density. Participants completed standardized questionnaires, and serum samples were screened for antibodies against Rift Valley fever virus, Crimean-Congo haemorrhagic fever virus, and Hantaan virus using a Luminex-based multiplex immunoassay. Logistic regression models were used to identify independent risk factors.

**Results:**

Among 2,019 participants, crude seroprevalence was 15.1 percent (95 percent confidence interval: 13.5–16.7) for Rift Valley fever virus, 10.8 percent (9.4–12.2) for Crimean-Congo haemorrhagic fever virus, and 2.2 percent (1.6–3.0) for Hantaan virus. Exposure to Rift Valley fever virus was higher in Matam than in Thiès, whereas exposures to the other two viruses were higher in Thiès. Older age and male sex were consistently associated with infection, and exposure to Crimean-Congo haemorrhagic fever virus was also linked to raw milk consumption and slaughterhouse work. During the study, the national sentinel surveillance system detected only one case each of Rift Valley fever and Crimean-Congo haemorrhagic fever, indicating a substantial cases under-detection.

**Conclusions:**

This study provides serological evidence of human hantavirus exposure in Senegal and confirms subclinical circulation of Rift Valley and Crimean-Congo haemorrhagic fevers. Our results suggest that routine surveillance is missing the vast majority of infections. These viruses circulate endemically within exposed populations, often in an asymptomatic or subclinical state, or manifesting with mild symptoms. This under-detection by the current monitoring system poses a significant challenge to the implementation of effective control strategies in endemic regions. This highlights the need to strengthen One Health surveillance to ensure early warning and public health preparedness.

## Introduction

Emerging and re-emerging zoonotic diseases such as Rift Valley Fever (RVF), Crimean Congo Haemorrhagic Fever (CCHF) and hantaviruses infection (HTN) pose major global health threats. Climatic and environmental changes, human encroachment into wildlife habitats, and intensified livestock movements are disrupting ecosystems, facilitating the spillover of animal pathogens to humans. Globally, up to half of human infectious diseases are zoonotic in origin, many maintained in wild reservoirs (1, 2). Understanding these dynamics is essential to prevent future outbreaks in vulnerable regions such as West Africa.

Rift Valley fever virus (RVFV) and Crimean-Congo haemorrhagic fever virus (CCHFV) are vector-borne RNA viruses transmitted mainly by mosquitoes (3–5) and ticks (6). Human infection typically occurs through bites or contact with infected animal tissues. Both viruses are endemic in West Africa and are increasingly associated with livestock infections (7, 8). In contrast, hantaviruses are rodent-borne viruses transmitted to humans primarily through the inhalation of aerosolized rodent excreta, and cause haemorrhagic fever with renal syndrome (HFRS) or hantavirus pulmonary syndrome (HPS), both associated with substantial morbidity and mortality (8, 9).

In Senegal, RVF was first reported during the 1987–88 epidemic near the Mauritanian border (10). Since then, RVFV transmission has been documented mainly in ruminants from the northern part of the country and in the Ferlo region. Subsequent outbreaks occurred in 2003 and 2013, with the latter marking the first detection of urban and peri-urban human cases in Dakar and Thiès (11, 12). Between 2020 and 2022, 13 human cases were reported in several regions of the country, including Matam, Saint-Louis, Fatick and Dakar, while a 2021 serosurvey in Matam revealed a human IgG prevalence of 20.8% and livestocks seroprevalence exceeding 60% (13–15).

Senegal has also reported widespread circulation of CCHFV in humans, livestock and ticks across multiple regions (15, 16). Between March and September 2023, eight confirmed human CCHF cases were identified in five regions, followed later that year by an additional eleven cases across seven regions (17, 18).

Serological evidence of human hantavirus exposure has now been reported in Senegal from samples collected between 2019 and 2022, marking the first identification in human samples with approximately 1 percent Hantaan virus (HTNV) IgG (19) alongside prior detection of Seoul orthohantavirus in wild black rats captured in 2012–2013 (20), highlighting the need for strengthened surveillance.

Early detection of zoonotic viruses with epidemic potential remains challenging due to asymptomatic subclinical infections, limited diagnostic capacity, and incomplete understanding of human and environmental risk factors. Despite recurrent outbreaks, few studies have systematically assessed human exposure and associated risk factors for multiple haemorrhagic fever viruses in Senegal. Data remain particularly scarce for transboundary pastoral areas, where close human–animal contact and seasonal livestock movements may facilitate viral spillover.

Accordingly, this study aimed to determine the seroprevalence of Rift Valley fever, Crimean-Congo haemorrhagic fever, and hantavirus infections in high-risk populations and to identify associated factors in both transhumant and sedentary communities of Senegal.

## Materials and methods

We conducted a repeated cross-sectional study between September 2022 and June 2024 to assess the seroprevalence of RVF, CCHF, and hantavirus infections in humans across different seasons and epidemiological settings in Senegal.

### Study sites

**Figure 1:**
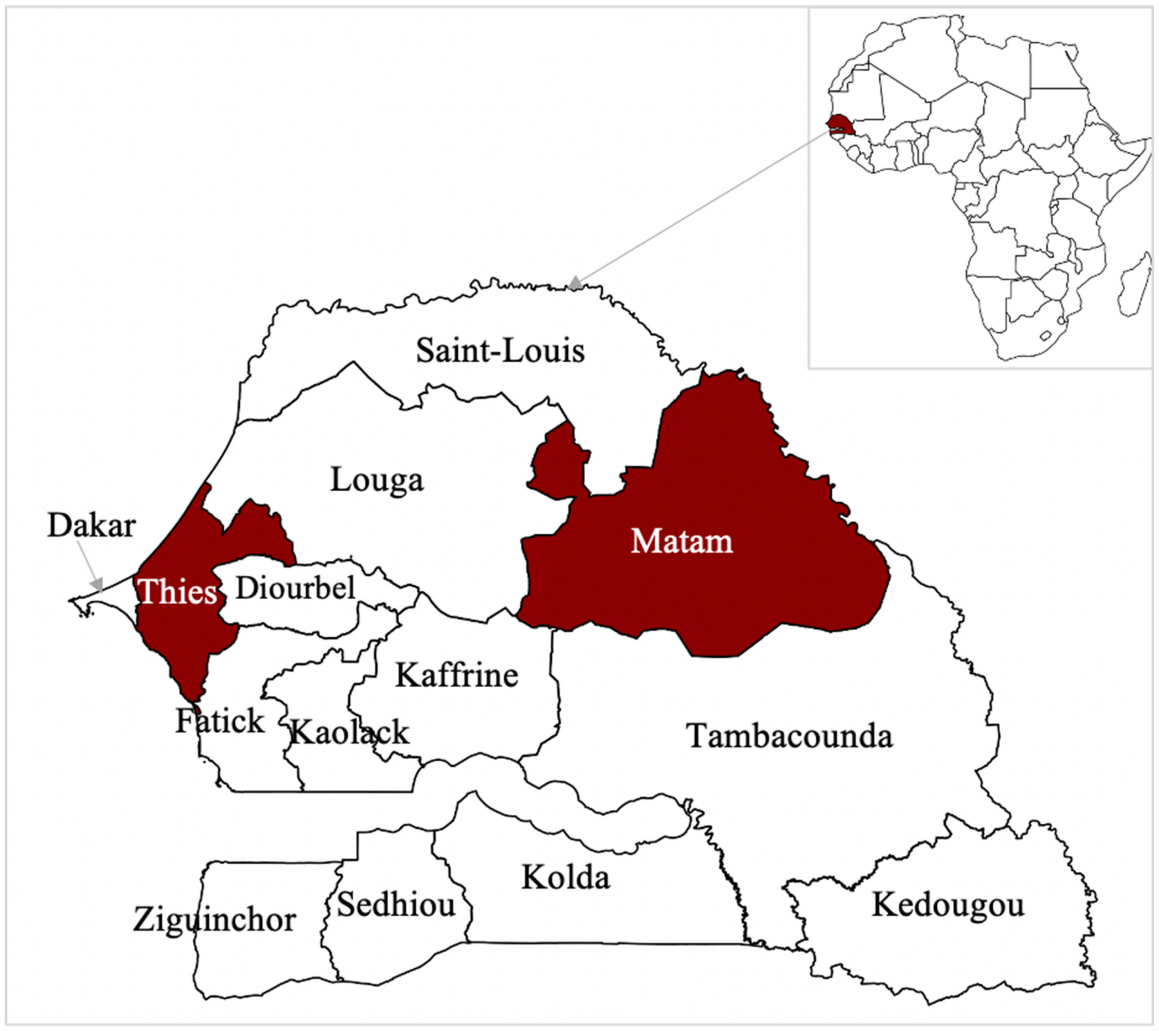
Study sites. Map of Senegal indicating selected study regions (in red). Other Senegalese regions are represented in white.

Two sites in Senegal were selected to compare transboundary and non-transboundary regions with differing ecological and epidemiological profiles (Figure 1). The Matam region, located in north-eastern Senegal along the borders with Mauritania and Mali, represents a transboundary pastoral ecosystem characterized by arid landscapes and extensive seasonal livestock movements. During the dry season, transhumant herders migrate southward in search of pastures, returning north during the rainy season (21).

In contrast, the Thiès region, situated in western Senegal, is a non-transboundary area with high human population density and substantial livestock holdings. It has a history of zoonotic outbreaks and is influenced by peri-urban farming practices that promote close contact between animals and humans. Recent studies have detected RVFV in local livestock (12) and CCHFV in both ticks and animals in the area, with human cases occurring within a 5 km radius (22). In addition, bat colonies known to harbor zoonotic viral pathogens are also present in the vicinity (23).

### Ethical considerations

Participants at high risk for zoonotic transmission in these regions were identified and invited to participate. All participants consented to participate in the study. For those younger than 15 years of age, a legal representative provided informed consent. The study was approved by the Senegalese National Ethics Committee for Research in Health (reference number N°000242/MSAS/DPRS/CNERS/SP, 07 September 2022).

### Sample size calculation

Based on IgG antibodies, a recent study conducted in Senegal in 2021 estimated a human prevalence of 20.8% (24). Based on this, with an absolute error of ±5%, a power of 90%, a sample size of 253 individuals was needed in each study site and each survey. This power and precision were selected to ensure reliable regional comparisons and to detect at least a 5 percent difference in seroprevalence between study sites or survey rounds. Overall, from September 2022 to June 2024, the total sample size targeted for both regions were estimated to be 2,024 individuals.

### Data collection and blood sampling

A cross-sectional study of asymptomatic individuals was conducted in Matam, in the north of the country, and Thies, in the west. The aim was to compare the Matam region, which has a transhumant population with significant movement of animals and people, with the Thies region, which has a more stable population but a high livestock density. The target population included individuals living in close contact with livestock, such as livestock farmers, slaughterhouse workers, and butchers.

For each participant, a whole blood sample was collected in a dry Vacutainer tube using standard venipuncture procedures, and a structured risk-exposure questionnaire was completed to capture demographic characteristics, occupational data, animal contact, and travel history. Blood samples were immediately stored at 4 °C and transported to the Institut Pasteur de Dakar for laboratory testing. Field investigation forms were checked for accuracy before data entry and subsequently stored in a centralized electronic MySQL database. The collected information included age, sex, occupation, residence, animal exposure, and travel history.

### Laboratory testing

Serum samples were screened for immunoglobulin G (IgG) and immunoglobulin M (IgM) antibodies against RVFV, CCHFV, and HTNV using a Luminex-based multiplex magnetic-bead immunoassay (MAGPIX, Luminex Corp). For each viral target, the nucleoprotein antigen was coupled to distinct microsphere sets to capture virus-specific antibodies. This multiplex assay was verified using animal models and known IgG-positive human samples (25, 26), and later validated for RVFV and CCHFV (23). This confirmed its suitability for large-scale surveillance in Senegal, showing good agreement with reference serological methods.

In brief, serum samples were heat-inactivated for 30 min at 37 °C and diluted 1:100 in PBST-SK (phosphate-buffered saline with 0.02 percent Tween 20 and 5 percent skim milk). The magnetic-bead mix was diluted 1:250 in PBST and dispensed into 96-well plates along with diluted samples, positive controls (known IgG/IgM-positive convalescent sera), and negative controls. Plates were incubated for 1 h, washed, and then incubated with anti-human IgG-PE or IgM-PE conjugates diluted 1:100 in PBST-SK. After final washing, 100 μL of PBST was added to each well, and fluorescence was read on the MAGPIX instrument.

Samples were considered positive when the mean fluorescence intensity exceeded 20 times that of the negative control, a threshold established through cross-validation with microneutralization assays on an independent Ghanaian cohort. As previously described, the MAGPIX platform was used here as a fixed-dilution qualitative screening assay for IgG and IgM detection. Because the assay does not include antibody titration curves, it does not allow quantitative interpretation of IgM/IgG ratios or timing of infection. Its intended purpose in this study is the identification of past exposure rather than dating of immune responses.

### Data analysis

Crude seroprevalence rates were calculated with the 95% confidence interval (CI). The 95% CI for seroprevalence were estimated using a binomial distribution.

We evaluated potential risk factors for RVF, CCHF and HTN, including region, sex, age group [(under 15], (15–45], (45–65], (65–98] years-old], season of sample collection (dry season, rainy season), occupation (breeders, student and/or teachers, housewives, medical staff, slaughterhouse staff and milk sellers, as well as other non-listed occupations), contact with wild animals, raw milk consumption and the use of protective equipment when handling animals. Fisher's exact test was used to compare the seroprevalence of RVFV (respectively CCHFV, HTNV) between the rainy and dry season.

Statistical analysis was performed for each disease using univariate binary logistic regression models. Significant variables from the univariate analysis were incorporated into the multivariable binary logistic regression analysis to evaluate the statistical significance of the association between the independent and response variables using adjusted odds ratios (AOR), 95% confidence intervals (CI) for AOR and *p*-values. A backward selection procedure was then applied to identify the variables that remained significant at the 0.05 level in the final model.

All statistical analyses and mapping were carried out using the R statistical language (25) version 4.0.4 within Rstudio (Version 1.2.1335).

### Syndromic sentinel surveillance network in senegal (4S network)

To complement serological findings, data from the national 4S (Syndromic Sentinel Surveillance) system were reviewed to identify confirmed human cases of RVF and CCHF reported during the study period. The 4S network, coordinated by the Senegalese Ministry of Health and the Institut Pasteur de Dakar since 2011, monitors epidemic-prone diseases through a network of sentinel health facilities across the country (24, 26).

## Results

Between 2022 and 2024, eight seroprevalence surveys were conducted. Four surveys were carried out at each site: two during the rainy season and two during the dry season (Table 1). A total of 38 sites (16 in Matam and 22 in Thies) provided 2,019 participants during this time period: 1,076 from Matam and 948 from Thies.

**Table 1:** HFVs seroprevalence and surveillance data in the Matam and Thies regions.

**Region**	**Season**	**Collection dates**	**Seroprevalence surveys (crude proportions)**				**4S Network surveillance**	
			**Collected (N)**	**RVFV (%)**	**CCHFV (%)**	**HTNV (%)**	**Number of RVF cases detected**	**Number of CCHF cases detected**
Matam	Rainy	Sept 22–Oct 06, 2022	251	17.62	8.20	0.00	1	1
		Sept 24–Oct 01, 2023	226	29.91	16.14	4.48	-	-
	Dry	May 22–30, 2023	267	16.33	9.16	0.00	-	-
		May 22–June 1, 2024	332	15.50	6.38	1.22	-	-
Thies	Rainy	Nov 09–20, 2022	260	14.29	15.52	2.63	-	-
		Sept 17–23, 2023	224	5.36	10.71	0.89	-	-
	Dry	Jun 04–11, 2023	254	15.35	13.39	3.94	-	-
		May 27–June 6, 2024	205	8.82	11.27	6.37	-	-
**Number of samples with co-infections**			**RVFV**+**/CCHFV**+**/HNTV**+	**RVFV**+**/CCHFV**+	**CCHFV**+**/HNTV**+	**CCHFV**+**/HNTV**+		
		Total	11	63	19	19		

Positive samples are noted as “+”.

Among the viral haemorrhagic fevers investigated, RVF was the most prevalent, followed by CCHF and HTN. The estimated crude seroprevalence was 15.06% (95%CI = 13.52–16.69) for RVF, 10.75% (95%CI = 9.43–12.18) for CCHF and 2.23% (95%CI = 1.63–2.97) for HTN.

For RVF, seroprevalence was significantly higher in Matam (19.27%) than in Thies (11.17%) (Fisher's exact test *p*-value < 0.001). For CCHF, seroprevalence was significantly higher in Thies (12.8%) than in Matam (9.55%) (Fisher's exact test *p*-value = 0.025). For HTN, seroprevalence was significantly higher in Thies (3.41%) than in Matam (1.34%) (Fisher's exact test *p*-value = 0.002) (Table 1). Co-infections between different viruses were also observed in the tested samples: 11 samples tested positive for RVFV, CCHFV and HNTV; 63 samples tested positive for FVRV and CCHFV; 19 samples tested positive for RVFV and HNTV; and 19 samples tested positive for CCHFV and HNTV (Table 1).

**Figure 2:**
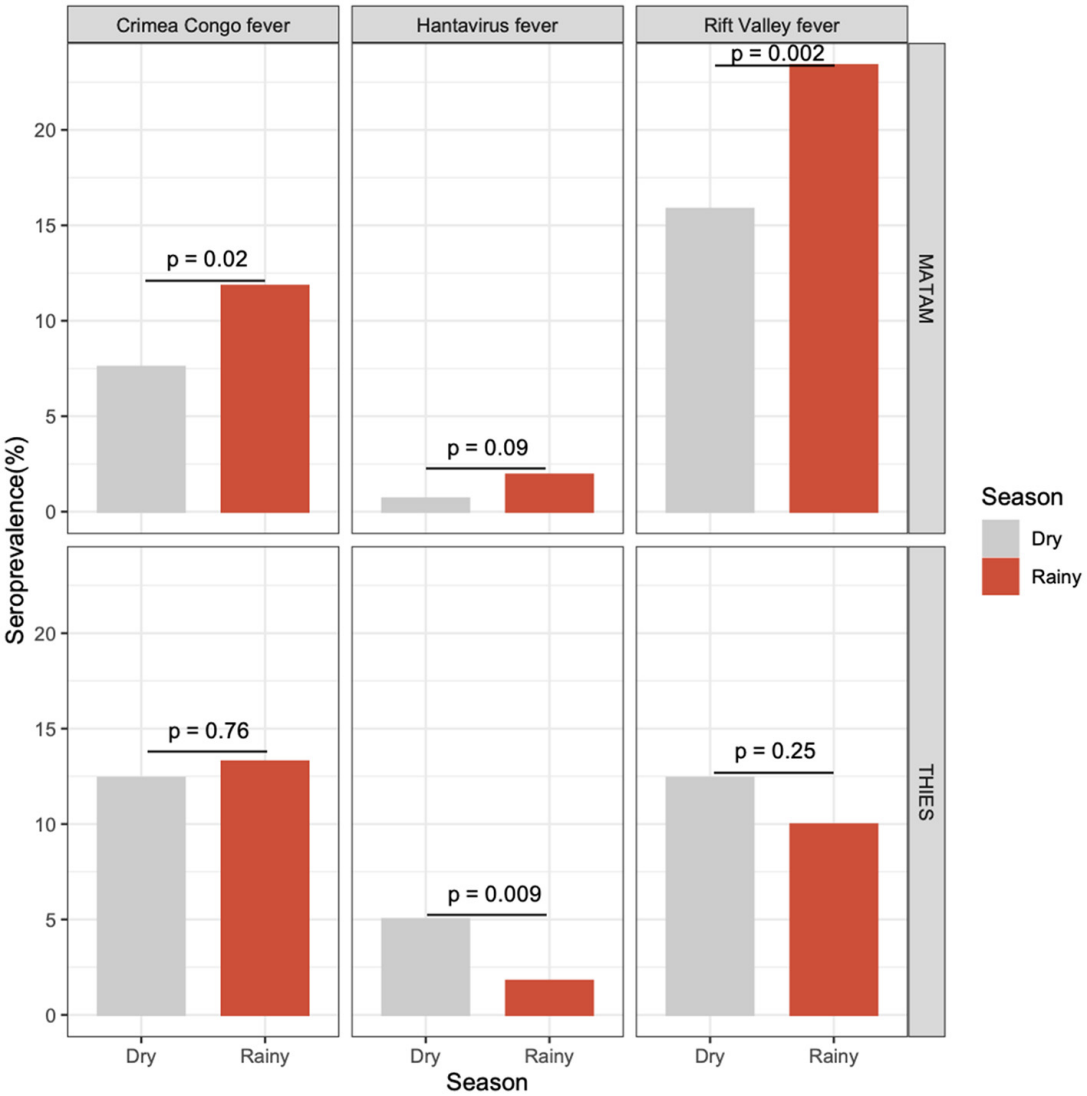
Seroprevalence comparison between rainy and dry season. Haemorrhagic fever viruses (HFVs) are represented in column. The first line panel represent seroprevalence for each HFVs in Matam. The second line panel represent seroprevalence for each HFVs in Thies. Gray bars represent the dry season and red one the rainy season.

Regionally, we observed that in Matam, seroprevalence was significantly higher during the rainy season than the dry season for RVF (*p* = 0.002) and CCHF (*p* = 0.02). However, in Thies, the differences in seroprevalence observed between the dry and rainy seasons were not statistically significant for RVF or CCHF. For HTN, seroprevalence was significantly higher in the dry season than in the rainy season in Thies (Figure 2).

**Figure 3:**
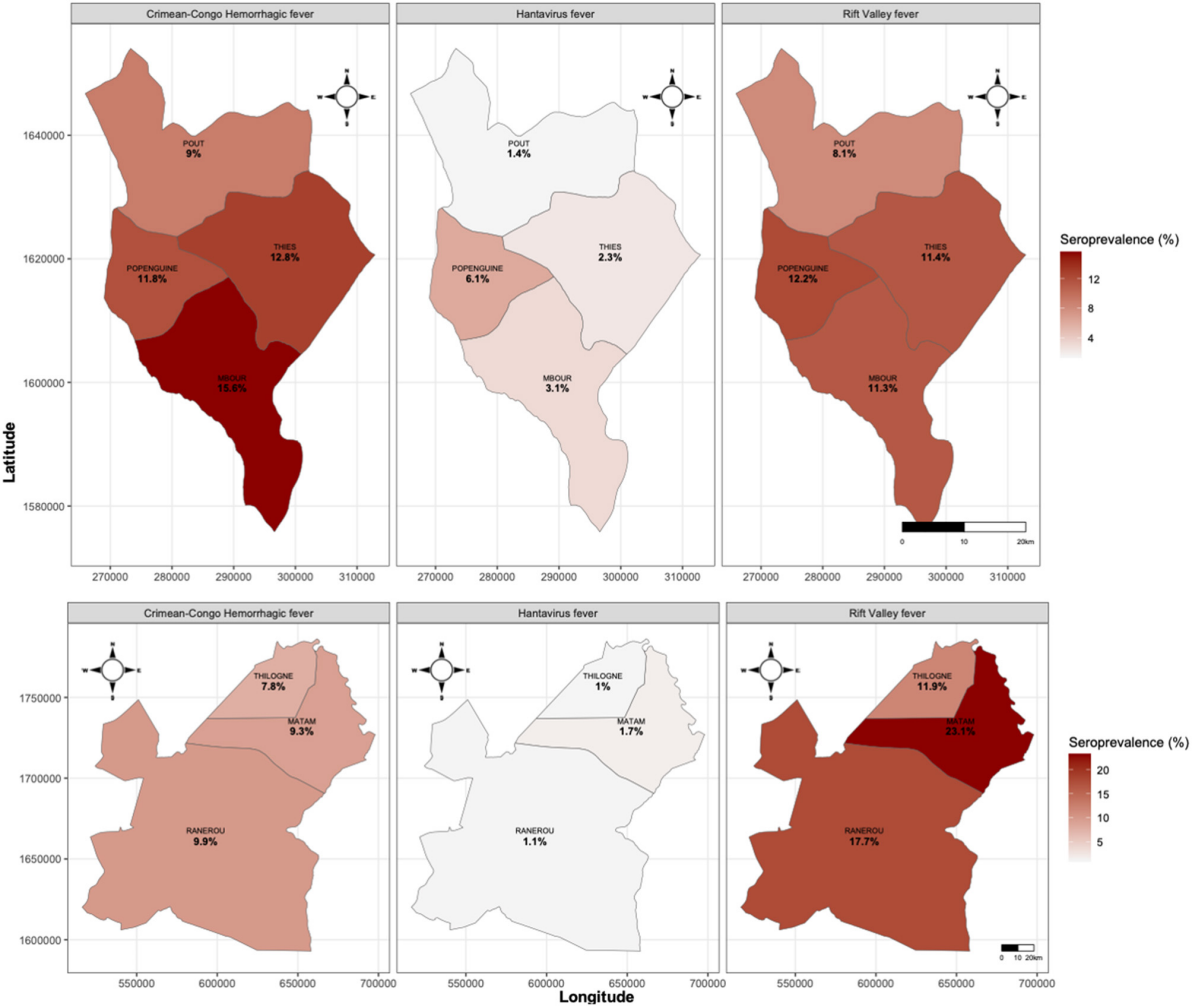
Spatial distribution of Haemorrhagic fever viruses seroprevalence at district level.

Regarding the distribution of seroprevalence by health district, RVF was the most widespread, being present in all surveyed districts. The highest seroprevalences were recorded in the Matam district (23.38%), followed by the Ranerou district (17.7%), the Popenguine district (12.2%) and the Thilogne health district (11.9%). The highest CCHF seroprevalence was found in the Mbour health district (15.6%), followed by the Thies health district (12.8%), the Popenguine health district (11.8%). For HTN, the highest seroprevalence values were particularly observed in Popenguine (6.1%), Mbour (3.1%) and Thies health districts (Figure 3).

For RVF, factors significantly associated with infection included gender, age group and region. During the entire study period, the odds of being infected by RVFV were higher among participants over 45 years old compared with those under 15 years old (AOR = 2.72, 95% CI = 1.79–4.13, p <0.001 for 45–64 years old and AOR = 2.95, 95% CI = 1.7–5.09, p <0.001 for over 65 years old). RVFV infection was 1.7 times higher in men than in women (AOR = 1.7, 95% CI = 1.3–2.21, p <0.001). Participants living in Matam region were 2.21 times more likely to be infected with RVFV than those living in Thies region (AOR = 2.21, 95% CI = 1.68–2.91, p <0.001) (Table 2).

**Table 2:** Univariate and multivariable factors analysis for RVFV infection.

**Variables**	**Labels**	**Number of collected samples (%)**	**Number of positive cases**	**Proportion of positive cases (%)**	**Univariate logistic regression**			**Multivariate logistic regression**		
					**Crude OR**	**95%CI**	***P*** **. value**	**Adjusted OR**	**95%CI**	***P*** **. value**
Sex	Female	1,008 (49.9)	126	12.5	-	-	-			
	Male	1,003 (49.7)	176	17.5	1.49	[1.16; 1.91]	0.002	1.7	[1.3; 2.21]	<0.001
	Missing	8 (0.4)	2	25						
Age Group	[0–15]	372 (18.4)	38	10.2	-	-	-			
	[15–45]	1,013 (50.2)	126	12.4	1.25	[0.85; 1.83]	0.257	1.32	[0.89; 1.98]	0.168
	[45–65]	457 (22.6)	102	22.3	2.53	[1.69; 3.77]	<0.001	2.72	[1.79; 4.13]	<0.001
	[65–98]	130 (6.4)	30	23.1	2.64	[1.55; 4.47]	<0.001	2.95	[1.7; 5.09]	<0.001
	Missing	47 (2.3)	8	17						
Region	Thies	943 (46.7)	102	10.8	-	-	-			
	Matam	1,076 (53.3)	202	18.8	1.91	[1.47; 2.46]	<0.001	2.21	[1.68; 2.91]	<0.001
Year	Year 1	1,032 (51.1)	156	15.1	-	-	-			
	Year 2	987 (48.9)	148	15	0.99	[0.78; 1.26]	0.939			
Season	Dry	1,059 (52.5)	149	14.1	-	-	-			
	Rainy	955 (47.3)	154	16.1	1.17	[0.92; 1.5]	0.198			
	Missing	5 (0.2)	1	20						
Profession	Others	375 (18.6)	60	16	-	-	-			
	Student–Teachers	299 (14.8)	29	9.7	0.56	[0.35; 0.9]	0.017			
	Breeders	609 (30.2)	112	18.4	1.18	[0.84; 1.67]	0.338			
	Housewives	503 (24.9)	69	13.7	0.83	[0.57; 1.21]	0.345			
	Medical-staff	76 (3.8)	7	9.2	0.53	[0.23; 1.22]	0.134			
	Slaughterhouse-staff	126 (6.2)	24	19	1.24	[0.73; 2.08]	0.429			
	Milk sellers	31 (1.5)	3	9.7	0.56	[0.17; 1.91]	0.356			
Contact with animal	No	64 (3.2)	7	10.9	-	-	-			
	Yes	1,914 (94.8)	291	15.2	1.46	[0.66; 3.23]	0.351			
	Missing	41 (2)	6	14.6						
Raw milk consumption	No	294 (14.6)	45	15.3	-	-	-			
	Yes	1,688 (83.6)	254	15	0.98	[0.69; 1.38]	0.909			
	Missing	37 (1.8)	5	13.5						
Use protective equipment	No	1,929 (95.5)	299	15.5	-	-	-			
	Yes	45 (2.2)	2	4.4	0.25	[0.06; 1.05]	0.059			
	Missing	45 (2.2)	3	6.7						

OR, Odds Ratio; 95CI, 95% Confidence Interval.

Analysis of CCHFV exposure showed significant associations with gender, age group, consumption of raw milk and certain occupations. Compared with women, men were 1.9 times more likely to be infected by CCHFV (AOR = 1.9, 95% CI = 1.39–2.61, p <0.001). Compared with participants younger than 15 years old, the odds of being infected by CCHFV were significantly higher among participants aged 45–64 years old (AOR = 3.27, 95% CI = 1.95–5.48, p <0.001) and those aged 65 years or older (AOR = 5.42, 95% CI = 2.96–9.93, p <0.001). Participants who reported consuming raw milk also had higher odds of infection (AOR = 1.85, 95% CI = 1.13–3.03, p <0.014). Occupational exposure further influenced risk, with significantly higher odds among slaughterhouse staff (OR = 2.27, 95% CI = 1.26–4.07, p = 0.006) and livestock breeders (OR = 2.26, 95% CI = 1.49–3.43, p <0.001) (Table 3).

**Table 3:** Univariate and multivariable factors analysis for CCHFV infection.

**Variables**	**Labels**	**Number of collected samples (%)**	**Number of positive cases**	**Proportion of positive cases (%)**	**Univariate logistic regression**			**Multivariate logistic regression**		
				**Crude OR**	**95%CI**	***P*** **. value**	**Adjusted OR**	**95%CI**	***P*** **. value**
Sex	Female	1,008 (49.9)	73	7.2	-	-	-			
	Male	1,003 (49.7)	144	14.4	2.15	[1.6; 2.89]	<0.001	1.9	[1.39; 2.61]	<0.001
	Missing	8 (0.4)	0	0						
Age Group	[0–15]	372 (18.4)	21	5.6	-	-	-			
	[15–45]	1,013 (50.2)	79	7.8	1.41	[0.86; 2.32]	0.172	1.4	[0.84; 2.33]	0.199
	[45–65]	457 (22.6)	78	17.1	3.44	[2.08; 5.69]	<0.001	3.27	[1.95; 5.48]	<0.001
	[65–98]	130 (6.4)	33	25.4	5.69	[3.15; 10.27]	<0.001	5.42	[2.96; 9.93]	<0.001
	Missing	47 (2.3)	6	12.8						
Region	Matam	943 (46.7)	117	12.4	-	-	-			
	Thies	1,076 (53.3)	100	9.3	0.72	[0.55; 0.96]	0.025	0.84	[0.62; 1.14]	0.264
Year	Year 1	1,032 (51.1)	113	10.9	-	-	-			
	Year 2	987 (48.9)	104	10.5	0.96	[0.72; 1.27]	0.765			
Season	Dry	1,059 (52.5)	101	9.5	-	-	-			
	Rainy	955 (47.3)	115	12	1.3	[0.98; 1.72]	0.07			
	Missing	5 (0.2)	1	20						
Profession	Others	375 (18.6)	32	8.5	-	-	-			
	Student-Teachers	299 (14.8)	13	4.3	0.49	[0.25; 0.95]	0.034			
	Breeders	609 (30.2)	106	17.4	2.26	[1.49; 3.43]	<0.001			
	Housewives	503 (24.9)	34	6.8	0.78	[0.47; 1.28]	0.325			
	Medical-staff	76 (3.8)	4	5.3	0.6	[0.2; 1.74]	0.342			
	Slaughterhouse-staff	126 (6.2)	22	17.5	2.27	[1.26; 4.07]	0.006			
	Milk sellers	31 (1.5)	6	19.4	2.57	[0.98; 6.73]	0.054			
Contact with animal	No	64 (3.2)	7	10.9	-	-	-			
	Yes	1,914 (94.8)	206	10.8	0.98	[0.44; 2.18]	0.965			
	Missing	41 (2)	4	9.8						
Raw milk consumption	No	294 (14.6)	20	6.8	-	-	-			
	Yes	1,688 (83.6)	190	11.3	1.74	[1.08; 2.8]	0.024	1.85	[1.13; 3.03]	0.014
	Missing	37 (1.8)	7	18.9						
Use protective equipment	No	1,929 (95.5)	203	10.5	-	-	-			
	Yes	45 (2.2)	4	8.9	0.83	[0.29; 2.34]	0.724			
	Missing	45 (2.2)	10	22.2						

OR, Odds Ratio; 95CI, 95% Confidence Interval.

In the case of hantaviruses, infection risk was primarily associated with age and region. Compared with participants under 15 years old, the odds of HNTV infection were higher among individuals aged 15–44 years (AOR = 34.53, 95% CI = 1.06–19.25, p = 0.041) and 45–64 years (AOR = 4.87, 95% CI = 1.08–21.92, p = 0.039). Participants living in the Thies region were 2.52 times more likely to be infected with HTNV than those living in the Matam region (AOR = 2.52, 95% CI = 1.29–4.89, p = 0.007) (Table 4).

**Table 4:** Univariate and multivariable factors analysis for HTNV infection.

**Variables**	**Labels**	**Number of collected samples (%)**	**Number of positive cases**	**Proportion of positive cases (%)**	**Univariate logistic regression**			**Multivariate logistic regression**		
				**Crude OR**	**95%CI**	***P*** **. value**	**Adjusted OR**	**95%CI**	***P*** **. value**
Sex	Female	1,008 (49.9)	19	1.9	-	-	-			
	Male	1,003 (49.7)	26	2.6	1.39	[0.76; 2.52]	0.286			
	Missing	8 (0.4)	0	0						
Age Group	[0–15]	372 (18.4)	3	0.8	-	-	-			
	[15–45]	1,013 (50.2)	24	2.4	2.98	[0.89; 9.97]	0.076	4.53	[1.06; 19.25]	0.041
	[45–65]	457 (22.6)	13	2.8	3.6	[1.02; 12.73]	0.047	4.87	[1.08; 21.92]	0.039
	[65–98]	130 (6.4)	3	2.3	2.91	[0.58; 14.58]	0.195	3.88	[0.64; 23.55]	0.141
	Missing	47 (2.3)	2	4.3						
Region	Matam	1,076 (53.3)	14	1.3	-	-	-			
	Thies	943 (46.7)	31	3.3	2.58	[1.36; 4.88]	0.004	2.52	[1.29; 4.89]	0.007
Year	Year 1	1,032 (51.1)	16	1.6	-	-	-			
	Year 2	987 (48.9)	29	2.9	1.92	[1.04; 3.56]	0.038			
Season	Dry	1,059 (52.5)	27	2.5	-	-	-			
	Rainy	955 (47.3)	17	1.8	0.69	[0.38; 1.28]	0.241			
	Missing	5 (0.2)	1	20						
Profession	Others	375 (18.6)	8	2.1	-	-	-			
	Student-Teachers	299 (14.8)	3	1	0.46	[0.12; 1.77]	0.261			
	Breeders	609 (30.2)	20	3.3	1.56	[0.68; 3.57]	0.295			
	Housewives	503 (24.9)	8	1.6	0.74	[0.28; 1.99]	0.553			
	Medical-staff	76 (3.8)	0	0	0	[0; Inf]	0.984			
	Slaughterhouse-staff	126 (6.2)	4	3.2	1.5	[0.45; 5.08]	0.511			
	Milk sellers	31 (1.5)	2	6.5	3.16	[0.64; 15.59]	0.157			
Contact with animal	No	64 (3.2)	1	1.6	-	-	-			
	Yes	1,914 (94.8)	44	2.3	1.48	[0.2; 10.92]	0.699			
	Missing	41 (2)	0	0						
Raw milk consumption	No	294 (14.6)	5	1.7	-	-	-			
	Yes	1,688 (83.6)	39	2.3	1.37	[0.53; 3.5]	0.514			
	Missing	37 (1.8)	1	2.7						
Use protective equipment	No	1,929 (95.5)	44	2.3						
	Yes	45 (2.2)	0	0						
	Missing	45 (2.2)	1	2.2						

OR, Odds Ratio; 95CI, 95% Confidence Interval.

Taken together, these results reveal widespread past exposure to RVFV, CCHFV, and hantaviruses in high-risk populations, despite the near absence of confirmed human cases through routine surveillance. Over the same period, the 4S network identified only one Rift Valley fever case and one Crimean-Congo haemorrhagic fever case in Matam, and none in Thiès (Table 1), suggesting that active transmission may be far more widespread and cryptic than indicated by routine case reporting.

## Discussion

This study provides a comprehensive overview of the circulation and exposure patterns of three major viral haemorrhagic fevers, RVF, CCHF, and hantavirus infection, across contrasting ecological settings in Senegal. The results reveal sustained and widespread exposure to these pathogens, with distinct regional and seasonal trends that underline their endemic presence and the ongoing risk of spillover to humans. Our findings revealed higher prevalence rates of RVF and CCHF in Matam than those reported by Sankhe and colleagues (27) in the same region. This discrepancy may be attributed to differences in sampling strategies. Our study targeted high-risk populations, whereas Sankhe and colleagues selected households at random.

The high prevalence rates observed in this study indicate historical exposure to VHFs in both regions, confirming that RVF and CCHF remain endemic in Senegal. Beyond the well-studied health districts of Matam and Thilogne, this work provides new insight into virus circulation in Ranerou Ferlo, a zone marked by intense cross-border pastoral mobility between Senegal and Mauritania. In Mauritania where RVF is endemic, eight epidemics have been documented since 1987 (28), with human cases and associated deaths also reported in Nouakchott (29, 30).

For RVF specifically, seroprevalence in humans was significantly higher in Matam than in Thiès (Table 2). This difference likely reflects contrasting population dynamics and animal movement patterns: Matam functions as a transhumance corridor with extensive seasonal migration of livestock and herders, whereas Thiès hosts a more sedentary population. Such movements have long been associated with increased exposure risk, as pastoralists and their herds frequently encounter infected vectors in endemic areas. Indeed, previous studies have shown that migrating herds exhibit higher RVF antibodies levels than sedentary ones (31).

Gender, age, and occupation were consistent determinants of exposure. Men were about twice as likely as women to be seropositive for RVFV and CCHFV, consistent with their greater involvement in livestock handling, herding, and slaughter activities (Tables 2, 3). Similarly, seroprevalence increased with age, indicating cumulative exposure over time to infected vectors or animals. These trends align with previous studies in West and East Africa that have linked occupational and behavioral patterns to zoonotic virus transmission.

The higher CCHFV seroprevalence in Thiès may be linked to ecological conditions favorable to tick vectors. The presence of the Bandia Reserve, which hosts abundant wildlife–livestock interfaces and high tick densities (11), could maintain a natural transmission cycle independent of livestock movement. In Bandia, Senegal, a study conducted from 1986 to 1988 found a prevalence of anti-CCHF IgG of 3.2% in the human population (32). Similar hotspots have been described in semi-arid zones of Africa, where mixed ecosystems sustain long-term virus circulation (33).

The results of our study showed a statistically significant association between CCFHV infection and the consumption of raw or unpasteurised milk. These results should be interpreted with caution, as transmission of the virus through milk is not certain. Indeed, Studies conducted in Turkey have not detected any CCHFV RNA in milk samples from domestic animals (34). However, unpasteurised milk has been suggested as a possible route of exposure to CCHFV, particularly in regions where the virus circulates in livestock (35). Furthermore, documents and guidelines relating to risk assessment specifically advise against the use of unpasteurised milk in regions where the CCHFV is present, as a precautionary measure (36, 37).

The significant increase in RVF and CCHF seroprevalence observed in Matam during the rainy season may be explained by the higher abundance of mosquito vectors during this period. This point is consistent with a study conducted in Tanzania, which reported the highest RVF seroprevalence during the rainy season (38). Peaks in mosquito activity have also been shown to coincide with months of heavy rainfall (39). Furthermore, the elevated RVF and CCHF seroprevalence rates in Matam could be attributed to the seasonal increase in livestock density, as transhumant herders migrate northward during the rainy season (20).

Although no confirmed clinical cases of Hantavirus have been reported in Senegal, our study confirms human exposure to hantaviruses, consistent with recent serological evidence from Sankhe et al. (19), who also identified IgG reactivity to HTNV using the same validated Luminex multiplex assay. In the present study, average IgG seroprevalence reached 3.41% in the Thies region and 1.3% in the Matam, suggesting low but measurable exposure in both regions. These results extend earlier observations of Seoul orthohantavirus detected in *Rattus rattus*, indicating that multiple hantavirus lineages, both urban and sylvatic, may circulate in Senegal. Environmental conditions in the Thiès region, particularly around Popenguine and the Bandia Reserve, where extensive rodent diversity has been documented (over 1,200 individuals captured between 1975 and 2012), may facilitate such maintenance cycles and sporadic zoonotic spillover to humans (40). It is important to note that the MAGPIX assay, as implemented in this study, is a fixed-dilution qualitative platform not used to infer the timing of infection. Our conclusions focus on the geographical distribution and magnitude of hantavirus exposure rather than on the age of immune responses. This approach aligns with global hantavirus sero-epidemiology practices, in which IgG detection alone is considered sufficient for documenting population-level exposure (41).

It is essential to emphasize that during the two-year period of data collection, the 4S sentinel surveillance detected one case of RVF and one case of CCHF. This weak detection via the 4S system indicates that surveillance only scratches the surface compared to the serological prevalence rates observed in our study. This lack of detection can be explained by the fact that clinical cases are reported by healthcare facilities. However, in rural and pastoral contexts, access to healthcare remains limited, and mild or uncomplicated cases often go unreported. This underscores the importance of community-based surveillance systems capable of detecting weak epidemiological signals and providing early alerts to public health authorities.

The need to reinforce surveillance is underscored by the ongoing RVF outbreak currently affecting Senegal (42). This situation highlights the urgency of strengthening national surveillance systems through an integrated One Health approach that systematically links entomological, veterinary, and human (including community-level) monitoring. Expanding this framework to include livestock serosurveillance, wildlife and mosquito vector data from Matam and Thiès would provide valuable insight into transmission dynamics and ecological risk factors for CCHF, RVF and hantavirus infections. Incorporating predictive modeling and climate-based early warning tools will also be essential to anticipate outbreaks, guide vaccination or vector-control strategies, and mitigate future epidemic impacts across Senegal and the wider subregion.

## Conclusions

This study demonstrates widespread IgG reactivity to RVF, CCHF and hantaviruses in Senegal, confirming their endemic circulation and under-recognized public-health relevance. High RVF and CCHF seroprevalence were observed in Matam and Thiès, particularly among individuals in frequent contact with livestock and animal products. Elevated RVF rates in Matam likely reflect seasonal pastoral movements, whereas higher CCHF exposure in Thiès may relate to tick-rich environments such as the Bandia Reserve. Infection risk was shaped by gender, age, occupation and behaviors such as the consumption of raw milk. Men, older adults, and those engaged in herding or slaughter activities showed greater exposure, reflecting cumulative and occupational risks at the human–animal interface. Evidence of hantavirus antibodies further suggests low-level but persistent circulation maintained by rodent reservoirs in peri-urban and sylvatic habitats. Our results suggest that routine surveillance is missing the vast majority of infections. These viruses circulate endemically within exposed populations, often in an asymptomatic or subclinical state, or manifesting with mild symptoms. This under-detection by the current monitoring system poses a significant challenge to the implementation of effective control strategies in endemic regions. These findings highlight the need to reinforce One Health surveillance by integrating human, animal, and entomological data with behavioral risk monitoring. Strengthened diagnostics, community-based surveillance, and predictive modeling are essential to anticipate and mitigate future zoonotic outbreaks in Senegal and the wider region.

## Data Availability

The raw data supporting the conclusions of this article will be made available by the authors upon request.

## Author disclaimer

The opinions, interpretations, conclusions, and recommendations presented are those of the authors and are not necessarily endorsed by the U.S. Army or Department of Defense. Research involving human subjects adhered to the principles identified in the Belmont Report (1979) and, unless classified as exempt, was conducted in accordance with an Institutional Review Board-approved protocol and in compliance with DoD, federal, and state statutes and regulations relating to the protection of human subjects. Research involving human subjects certified as exempt was conducted in accordance with the conditions specified in connection with an Exemption Certificate. The use of either trade or manufacturers' names in this report does not constitute an official endorsement of any commercial products. This report may not be cited for purposes of advertisement.
